# Facebook as communication support for persons with potential mild acquired cognitive impairment: A content and social network analysis study

**DOI:** 10.1371/journal.pone.0191878

**Published:** 2018-01-29

**Authors:** Aboozar Eghdam, Ulrika Hamidi, Aniko Bartfai, Sabine Koch

**Affiliations:** 1 Health Informatics Centre (HIC), Department of Learning, Informatics, Management and Ethics (LIME), Karolinska Institutet, Stockholm, Sweden; 2 Division of Rehabilitation Medicine, Department of Clinical Sciences Danderyd Hospital, Karolinska Institutet, Stockholm, Sweden; Macquarie University, AUSTRALIA

## Abstract

**Introduction:**

Social media has the potential to increase social participation and support for the well-being of individuals with chronic medical conditions. To date, Facebook is the most popular social medium for different types of communication. However, there is a lack of knowledge about the potential use of Facebook as a means of communication for persons with potential Mild Acquired Cognitive Impairment (MACI), a non-progressive mild cognitive impairment after an acquired brain injury. The aim of this study was to explore how persons with potential MACI, specifically persons with perceived brain fatigue after brain injury, communicate through Facebook, to classify the content of the communication and to visualize the frequency and types of interactions.

**Methods and materials:**

A social network analysis of the interactions between members’ and a qualitative content analysis of a whole year’s communication of a public Facebook group for Swedish speaking persons (1310 members) with perceived brain fatigue after an illness or injury to the brain were performed.

**Results:**

The results showed how members use social media technology and Facebook as a means for communication and support for their condition. Individual group members showed very different patterns of communication and interactions. However, for the group as a whole, the most frequent topics in their communication were related to *informational support* and *banter* in posts, and *socialization* in comments. The findings also showed that the majority of members only communicated with few other members and had few direct communications. The most used communication feature of Facebook was likes in form of “thumbs-up”.

**Conclusions:**

This study indicated that social media and in this case Facebook is used for communication and social support by persons with potential MACI, and revealed that their communication behavior is similar to the healthy population. Further studies relating specific cognitive problems of the participants to the use of social media would provide more reliable results for this specific group.

## Introduction

One of the leading causes of disability in many developed countries is brain injury [1]. Mild Acquired Cognitive Impairment (MACI) is a term used to describe persons with non-progressive Mild Cognitive Impairment (MCI) acquired from a brain injury. They can have multiple cognitive (e.g. memory impairment) and/or somatic (e.g. headaches, fatigue) in addition to mild physical disabilities and their access to rehabilitation services to improve their affected skills is limited [2]. One approach that can be considered for further developing treatment of MACI is information and communication technology (ICT) and based on previous findings from Sweden [3], the most used types of ICT services are communication services. In Sweden over 70,000 persons suffer from brain injuries every year and the most common causes are stroke and head injuries [4]. About 80% of these persons suffer from mild impairments[4].

A serious illness affects various aspects of an individual’s life such as communication, self-care, social relationships, employment and mobility [5]. A person with a chronic condition is in great need of different kinds of social support on a long-term basis [6]. Previous research has shown that social networks in general may improve disease management for persons with chronic conditions, suggesting that online social networks have a great potential to improve health care [7–10]. Therefore, one of the biggest benefits of online social media is social support.

### Internet and social media

The use of the Internet and especially social media as a means for communication has increased over the past decades [11]. Social media has changed how health consumers and health care providers communicate [11,12]. McGowan and coworkers have shown that the use of social media in health care may be an efficient way to share up-to-date medical knowledge with others within a community and improve the quality of patient care [13]. Due to the increase of interest in online social support, many researchers have been investing in understanding the contents, motivations and the effects from their use and the interest in interactive consumer health technology applications has also increased [14,15].

Today, gaining knowledge and sharing experiences with others in similar situations are common phenomena in social media. Based on the results of a national survey that we have conducted recently, one of the most important types of support for persons with mild brain injury is communication services and especially “social interaction and relationships with families and friends” [3]. Sweden is one of the top countries among European individuals. According to Eurostat, in 2016 about 94% of Swedish households have access to the Internet and 91% of the population use the Internet at least once a week [16].

Facebook is the largest social networking site in the world with 1.65 billion monthly active users on average from which about 92% access it through mobile devices [17]. Generally, people use Facebook as a means for communication with relatives and friends and other people with similar interests [18]. For people with common interest, the group function is useful to share their opinions and communicate on the topic or purpose of the group. Users can join or create new public or private groups and post or share content on the group’s wall. The group’s privacy settings are controlled by ‘‘administrators” who have the ability to manage the content in addition to invite or approve the membership of new users. The content of public groups is visible to any user of the Internet even to any non-Facebook user.

### Social media for supporting patients with mild acquired cognitive impairment

Communicating with people in similar situations is helpful to cope with daily life as it reduces feeling of loneliness and isolation when dealing with a rare condition [19]. Generally, sharing experience of having the same condition as informational support in addition to socializing seems to be beneficial for persons with chronic conditions [20]. There is a socializing need for persons with MACI but since performing physical socializing requires so much energy due to problems with concentration and loosing track in a conversation, social activities appear to be more difficult in real-life situations. For persons with MACI, physical socializing decreases after brain injury and people get lonelier but the need to socialize still remains [21]. Therefore, social media would be an ideal solution since its use can be regulated depending on the individual’s condition and at one’s own pace. The long distance between individuals with MACI may hinder face-to-face conversations but the online communication would ease general accessibility and increase inclusiveness [22].

Today, many disease-specific groups exist on Facebook, people share various health issues in this platform [23]. and it became popular as a support-seeking- providing tool for patients with breast cancer and HIV [24,25]. For persons who are dealing with their situation by themselves, in addition to socializing, social media could be used for gaining knowledge from others as well as sharing experiences [26]. These kinds of social interactions among persons with a similar condition could also be beneficial for the social well-being of persons with MACI. Based on our previous research, the amount of persons with MACI who use Facebook is rather remarkable despite the reported cognitive problems [3]. Given the higher risk for a significant decrease in their friendships and social support and due to the large number of persons with mild brain injury, providing face-to-face social support may be problematic as a result of limitations in mobility, access, or communication. Therefore, online support groups may be a particularly useful alternative for these individuals [6].

Despite the potential value of online support, very little is known about how people with different health issues use Facebook groups and there is a lack of research examining social networking sites in the context of MACI [27,28]. To our knowledge, no studies on the use of Facebook groups as a means of providing social support for persons with brain injuries have been published. This study aims to explore how persons with potential MACI, specifically persons with brain fatigue after brain injury communicate through Facebook, to classify the content of the communication and to visualize the frequency and types of interactions.

## Methods and materials

The main methods for this study were content and social network analysis of communication within a Facebook group for Swedish speaking persons with brain fatigue after brain injury.

All content of the Facebook group’s timeline between September 1, 2014 until August 31, 2015 was collected. In October 2015, the group consisted of 1310 members. To reduce the ratio of potential missing data in the collected data set and to avoid seasonal effects on individuals’ behavior, the time window was set to a whole year [29]. The data was collected from users’ interactions in the group such as posts, written comments to posts or acknowledged “likes” to posts.

The researchers collecting the data presented themselves in the Facebook group as “researchers who would observe the group”. Individual informed consent was not acquired as the data was collected retrospectively. The approval of the study was obtained from the Regional Ethics Review Board (EPN) in Stockholm (2015/1284-31/5 October 01, 2015). The Regional Ethics Review Board (Regionala etikprövningsnämnden—http://www.epn.se/) has the task to vet cases within the field of medical science (medicine, pharmacology, odontology, the science of health care and clinical psychology). The secretariats of the regional boards are situated at the different universities in Sweden.

### Data collection

In this study Netvizz v1.25 was chosen as the data collection software. Netvizz is a Facebook application (data access via the Facebook API) that allows researchers to collect anonymous data from open (Public) Facebook groups [30]. The data collected by Netvizz was obtained in comma separated value (CSV) file and graph data file (GDF) format. This data collection was performed without any interaction with the Facebook group’s members and no attributes from the participants´ Facebook profiles (e.g. name, usernames, email, gender, etc.) were collected. Regarding the anonymization and to have unique identifiers, the participants were given automated anonymous Ids by the data collection software. The CSV files contained the texts and symbols in the posts and comments. The GDF files had tables with relational data on the members´ involvement in communicative-interactions.

### Data analysis

#### Content analysis

To understand the members’ contributions to the Facebook group, contents of the posts and comments were analyzed using a direct content analysis approach in which the initial starting categories and definitions for the themes were retrieved from literature (Table 1) [31]. The content analysis began with repeated reading of all posts and comments by the first and second authors. Basically the content was categorized into social supports and others. For social supports the posts and comments were grouped based on the initial codes retrieved from typology by Cutrona and Suhr’s [25,32]. The five broad categories for social support according to the Social Support Behavior Code (SSBC) developed by Cutrona and Suhr are defined as “informational”, “emotional”, “esteem”, “network”, and “tangible support” [32]. Table 1 shows the Cutrona and Suhr definitions for social support categories.

**Table 1:** Definitions of social support categories according to [32].

Social support categories	Definition
Informational support	Providing information such as suggesting courses of action or guidance for coping with illness-related challenges.
Emotional support	Expressing emotion or support the emotional expressions of the recipient.
Esteem support	Validating the recipient’s self-concept, importance, competence or rights as a person.
Network support	Articulation, expanding or deepening the structural connections an individual possesses.
Tangible assistance	Providing or offering to provide specific material aid or services to assist the recipient.

For other categories, the initial codes were retrieved from the Gaysynsky et al. study about analyzing a Facebook group for young adults living with HIV [25]. The definitions for other categories are shown in Table 2.

**Table 2:** Definitions of other categories according to [25].

Other categories	Definition
Expressions of gratitude	Thankfulness to another member of the group or the group as a whole.
Offering congratulations	Expression joy or acknowledgment of the recipient’s achievement or good fortune.
Administrative/engagement in group	Administrative engagements.
Banter	Messages that include humor or nonsense.
Socializing	Discussion of interacting outside the group environment, greetings (e.g. birthday or holiday wishes), invitations to events, photos and videos of the group, news about personal achievements or milestones, etc.
Group cohesion	Communication on how members feel about the group.
Negative interaction	Disrespectful or sarcastic comments directed at other participants or statements that express being hurt.
Community protection	Aim to maintain an atmosphere of support or enforce group norms.
Non-verbal cues	Expressions of non-verbal such as facial expressions and actions.
Miscellaneous	Statement which is not applicable to other codes.

During the content analysis process additional categories emerged that were classified by the authors of this paper. (Table 3)

**Table 3:** Definitions of additional categories as defined by the authors.

Additional categories	Definition
Advertising	Indirect or direct advertisement.
Questions	Questioned asked within the group which could not be coded according to any other of the codes.
Own comment	Comments on their own posts.

In addition to the posts and comments, the likes were counted. Furthermore, the context of a particular post or comment (i.e. content that precedes or follows the message of interest) was used to interpret the intention of the message, categorization and to see if the group member was seeking or providing support.

Microsoft Excel was used as a tool for organizing and coding the collected data. The first and second authors performed the analysis process and validated (to evaluate the correctness of the assigned categories) through discussion with the other authors.

#### Social network analysis

One method for measuring relations, associations and flows between different information and knowledge entities that can reveal patterns of the activities within social media groups is social network analysis (SNA) [33,34]. Generally, a SNA map consists of nodes (persons) that are linked to show the relationships and flows (information) between them.

A social network analysis was performed to analyze and visualize the distribution of the group members’ involvement in information exchange. In the Facebook group, participants are conceptualized as actors (nodes) and their comments and likes to each other’s posts are incorporated as the relations between them. In a social network, the nodes with more relations to others, are less dependent and more influential. Therefore, they may have alternative ways to satisfy needs [35,36]. The number of nodes that are in relation with one node, is defined as ***degree centrality*** and the number of interactions (comments on, or likes to posts) with each node is defined as ***weighted-centrality***. Also, the comments and likes around the posts between two unique nodes are defined as ***edges***. In this study, the location/degree centrality of nodes is evaluated and visualized to provide an understanding of the network and its members.

In order to provide the drawing of social network graphs as well as calculations of social network concepts, Gephi 0.8.2 beta software was used. Gephi is an open-source software that provides the opportunity to interact with the graph drawing algorithms [37]. Gephi provides several force-directed algorithm alternatives and visual representations of the data. The decision on choosing the graph drawing algorithm is a subjective task but it is advised to try all of them to be able to present the most comprehensible graph [38]. For constructing and to be able to modify the density of the visualization setting of the graph, the ForceAtlas2 algorithm was used. This algorithm was continuous and it was stopped when relatively stable and clear patterns were perceived.

## Results

**Fig 1:**
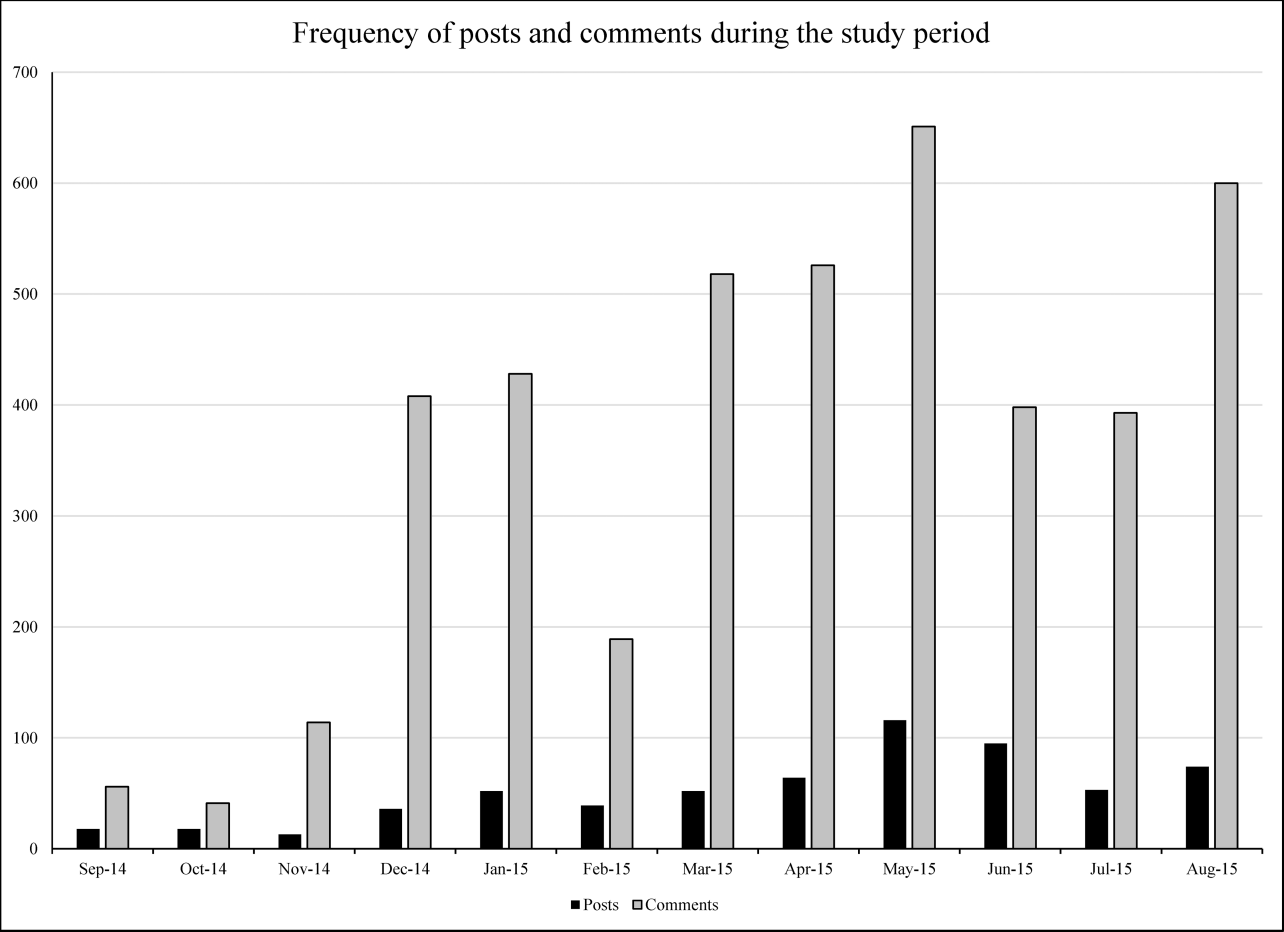
Number of posts and comments during the study period.

During the data collection the contents that appeared on the Facebook group’s timeline one-year period were collected. Fig 1 shows the numbers and frequency of posts and comments during this one-year time period. The maximum amount of posts and comments occurred during May 2015. Within the time window 1092 (83%) unique active members’ contributed to the Facebook group with 630 posts, 4323 comments and 10187 likes on posts. The amount of self-comments (comments on own posts) was 23%. 218 members did not have any interaction in the group.

### Content of the communication

Content analysis was applied to all posts and comments. Table 4 shows the incidence of the main interaction categories for posts and comments.

**Table 4:** The incidence of posts and comments based on main social support categories.

Categories		Posts (%)	Comments (%)
**Support**	Informational	41.3	26.3
	Emotional	9.4	20.4
	Esteem	4.9	14.3
	Network	1.6	0.4
	Tangible assistance	0.3	0.3
**Others**	Banter	13.7	0.9
	Socializing	13.3	4.9
	Expressions of gratitude	7.3	2.1
	Advertising	2.4	0.0
	Community protection	1.6	1.2
	Administrative/engagement in group	1.1	0.0
	Miscellaneous	1.1	0.1
	Non-verbal cues	0.8	3.2
	Questions	0.8	2.0
	Group cohesion	0.3	0.8
	Negative interaction	0.2	0.3
	Offering congratulations	0.0	0.1
	Self-comment	0.0	22.6

The majority of posts (57%) and comments (62%) were categorized as different types of social support. From 362 posts in the social support categories, most of them (72%) and a large amount of comments (43%) were peer to peer exchange of “informational” social support. Only 17% of social support posts were seeking support and 40% were providing support. Regarding the other categories, “banter” in posts and “socializing” in both posts and comments were the most frequently applied codes (excluding self-comments) (Table 4).

The informational social support comments and posts were mostly about the members’ health related problems or sharing an experience of having a health condition and strategies about how to deal with it (Table 5). Despite clinical differences, expressing their experiences made them feel less alone since they had a common empathic feeling. However, in some cases, the group members lacked the required medical knowledge to address specific questions or issues (such as adequate medication). In general, the group platform had an open learning atmosphere. As shown in Table 4, advertisement and negative interaction were quite rare in this group. The members had very few posts and comments coded as negative interactions (e.g. benefits of narcotic drugs).

**Table 5:** Exemplifying quotations from posts and comments.

Example post	Quotations
1	[Theme: Support through new strategies]
	Post by A: “I have chronic headache / stress headaches and has had it for about 6–8 years. Do you know any doctor in Sweden who is good at heads and its problems? Would do anything now to get some help!”
	Comment by B:”I have had migraine that was so powerful that the stomach contents came up. Not had a single attack the last 5–6 years, but however, never had a doctor who helped me. I got help from a physiotherapist, three treatments were enough:) The best thing I have done:)”
	Comment by A: “What kind of treatment was that?”
	Comment by C:”The physiotherapist makes treatments that help the body heal itself. He has helped us with many things in our family:)”
	Comment by A: “Thanks for the replies!”
2	[Theme: Support through new strategies]
	Post by A: “If you already have headache and dizziness when you wake up, how to get started in the morning?
	Comment by B:”Massaging the eyes and temples. It tends to drop a little then.”
	Comment by C:” Meditation? Relaxation Exercise? Hope the day will be better!”
	Comment by D:”Coffee.”
	Comment by E:”Hold on and hope that the next day starts better; -)”
	Comment by A: “Thank you all!”
3	[Theme: Support through new strategies]
	Post by A: “Now we put asthma also on top of all the other crap we suffer from. Sure there is medicine for it so you can feel better but still. Feel it’s enough now. Only hope my doctor prescribes medicine soon and hope it helps.”
	Comment by B:”Exercise is good for asthma!!”
	Comment by C: “Pity! But curable!!”
	Comment by A: “Yes, feels no fun but hope that medicine is beneficial so I can start training a little again.”
	Comment by E:”Sad!”
	Comment by F:”It can be really good with the right medications. I have a number of different diseases and I think that's pretty damn dull. “
	Comment by G:”Sad but you can treat it!!”
	Comment by A: “The exercise is good but at the moment, it does not work. Needs nothing and is being taking care of one by one. First examined the heart to rule out that it was because of it, since a spirometry was made on Friday and then they said it is asthma. . . “
	Comment by H:”Hugs! ♡”
	Comment by I: “In any case, I’m glad it was not anything serious. ☺”
4	[Theme: Support through new strategies]
	Post by A: “Today was a dangerous day. Was putting on the stove when I should boil water in the kettle. . . Ugh. Anyone having strategies for such days?”
	Comment by B: “check on the cat pictures on FB, but no more demanding activity. Alternatively lying in bed and do mindfulness exercises, so as to focus on where in the body it feels like to breathe and keep focus directed there. <3”
	Comment by C: “I have got a kitchen warner from the municipality. The stove goes out after half an hour! Great!”
	Comment by A: “Ok, thank you!”
5	[Theme: General support from other group members]
	Post by A: “I felt good today despite the rain and bad weather. Wish me such a good day tomorrow also thanks *** Then we were 2 * fixed both my makeup and had lunch with a friend, but know that tomorrow will not be as good. But hope one can.”
	Comment by B: “Happy for you that you felt good yesterday. Although the morning did not start so well, so the afternoon can get better. :) Otherwise, you try to enjoy the peace and quiet today. As long as you get it.”
	Comment by A: “Thank you! my whole family is a fine support. But it is most mother who helps me in different ways. :)”
6	[Theme: General support from other group members]
	Post by A: “Feel so bad. Employment Office just called and was unbelievable unpleasant and kept the pressure on me. Just want to give up everything. Cannot cope to feel like this and also to not get any understanding. Do not want to be here. . .”
	Comment by B: “Strength Hugs!”
	Comment by C: “It will feel better again. . . Oh here we understand you <3”
	Comment by A: “Thanks **”
7	[Theme: Support through sharing an understanding]
	Post by A: “How do you usually deal with your tiredness? I need advice. *”
	Comment by B: “Me too! I'm doing body scanning, guided meditation and powernaps.”
	Comment by C: “I have a structured life. Wake up the same time with the help of the alarm clock, sleep at certain times of the day, all punctuated. Then I feel the best, life is not much fun, but works well.”
	Comment by D: “Arrangement and reason, picked up at home. Guided meditation and yoga 2–3 times a week. Trying to structure life like C, but easily forget the time. . . ☺”
	Comment by E: “Yes, these with structure and order and I think also. It works quite well now that I'm not working. I start to work this fall and we'll see how I will feel then.”
	Comment by F: “Fatigue cannot go away (for me). Agree with previous posts, regular life routines are necessary. Do it as you can and be sure to take breaks. The worst thing for me is the day when it is expected to run from one to the other [things]”
	Comment by G: “Regular sleeping times. Avoids intensive environments if possible.”
	Comment by H: “It's really superficial, despite a busy life, when I cannot sleep either day or night, and as you say F, you cannot restrain your brain fatigue when your house is full of sounds, voices, etc. It's incredibly tough to live right now and I wish that I "learned" / find a way that works in everyday life but unfortunately “
	Comment by I: “It's true that you cannot skip the fatigue but the body feels better (body, psyche, a little clearer think, do not lose as many words, etc.) I do not sleep and I'm unwilling and not always so fit ☺ but I feel as tired. I have diagnosed ME/Cfs because I'm not just a few days late, I will not get out of bed for several days. If you regularly sleep, your mental health is better. And it's important to keep the mood up so it does not get even more difficult and mentally bad beyond what you already feel. “
	Comment by J: “Rest and just relax.”
	Comment by K: “I usually go for a walk without a phone by myself and gladly same round each time where I do not meet so much people. I sleep in this fantastic sleeping system! Link to a website”
	Comment by L: “Silence! sh sh sh. . . .”
	Comment by M: “Tired since last October now. Tired of fatigue. I check that I take micro pauses, a long pause in the middle of the day and if possible, a nap in the afternoon. I check that I have eaten, drunk, and if it is rather hot, eaten a little salt and drunk water”
	Comment by N: “If I get the anxiety of too much fatigue, I lie and breathe according to mindfulness.”
	Comment by O: “Good practice, mindfulness and health care system accept their cost and that there are good and bad days—it is difficult and every day is a challenge!”
	Comment by P: “Hard scheduling of rest and activity.”
	Comment by Q: “Silicone ear tips and noise cancelling headphones. I always have with me”
8	[Theme: Support through sharing an understanding]
	Post by A: “I am right now at the xx hospital for radiotherapy and the first production of my mask. I see the first mask in the flat state. …Following on to the computer tomography / CT with the mask on. An extended X-ray. Ended with radioactive tracers injected …_ and imaging with PET / CT camera. I was stuck with the mask for 20 minutes for the first time_ panicking feeling. Scans of the body. What happens in the camera hope that nothing more is seen of tumors and cancer!
	Comment by B: “Many of us are keeping our fingers crossed for you <3”
	Comment by B: “Yes, we are many!”
	Comment by C: “Fingers crossed!”
9	[Theme: Lack of medical knowledge to address specific questions or issues]
	Post by A: “How do you usually deal with your tiredness? I need advice.”
10	[Theme: Lack of medical knowledge to address specific questions or issues]
	Post by A: “Do you get better sleep if you take sleeping pills? Are you better off to sleep more? Is there anyone who has experience in sleep medicine for brain fatigue? Thanks in advance!”
	Comment by B: “I have eaten melatonin for over 1 year and may lay awake all night if I do not take any tablets. So need help falling asleep. But check after sleeping meds with substances that are already present in the body in order to fall asleep.”
	Comment by A: “Thanks for your reply, it must be awful to lie awake all night! Need to read more!”
	Comment by C: “Hello I eat Imovane 7.5mg if needed. Have managed to cut down on the intake of Imovane to perhaps 2–3 nights/week instead of 5–6 nights/ week. Check with your local brain injury center!”
	Comment by D: “The first year, it became a habit to not fall asleep before 3 am at night. But it is so nice to sleep! Now, last month I have eaten anti-depressives against my headache and back pain. O, it makes me very tired. To have been able to sleep 10-12h every night I ate them.”

### Frequency and types of interactions

**Fig 2:**
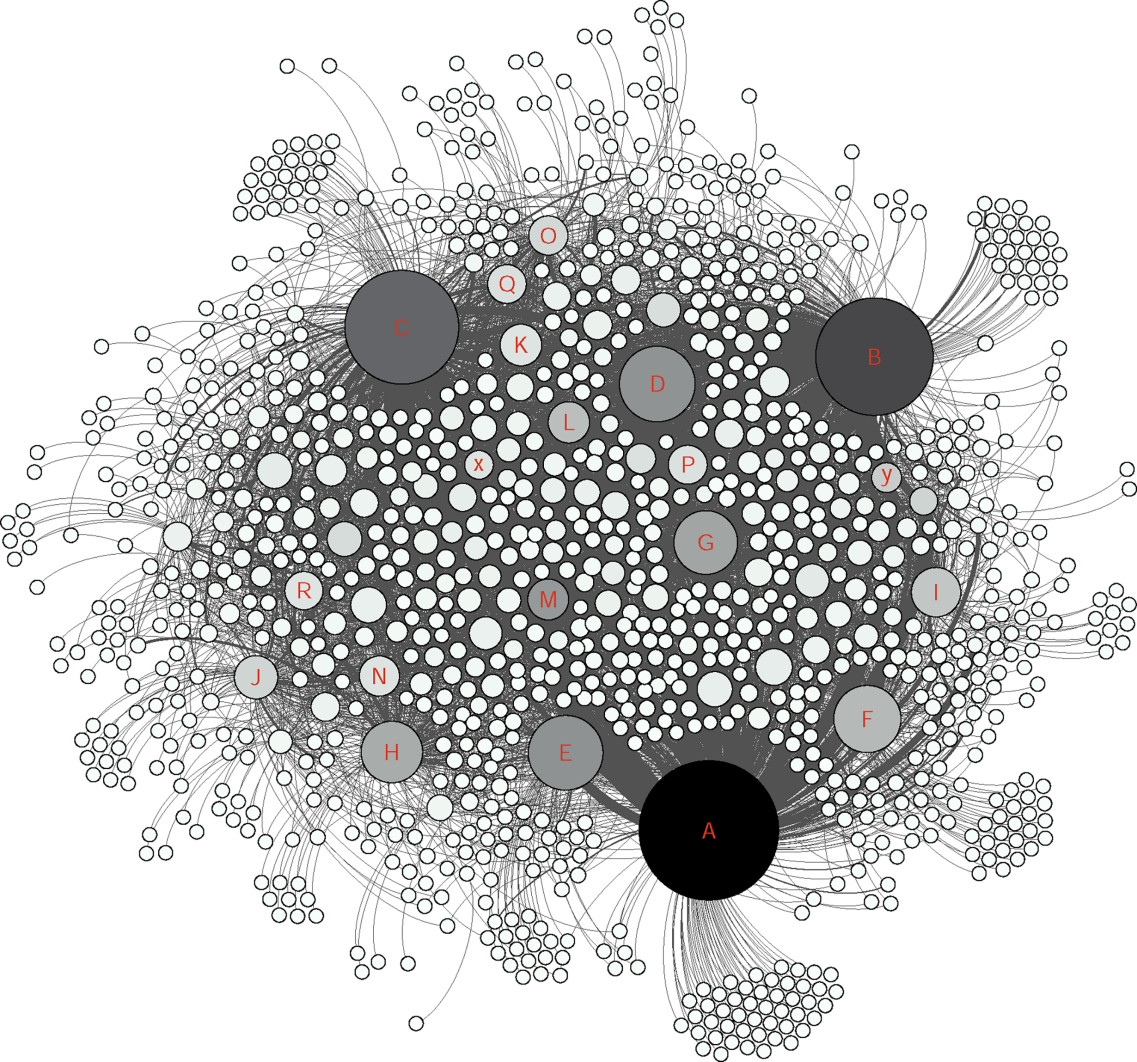
The whole network graph. The size of each node represents the degree centrality and the darkness indicates weighted-centrality. The marked nodes A-R are well-connected and very-well-connected nodes in the network. The x and y nodes are two other active nodes who just made “comments” and “likes”.

**Fig 3:**
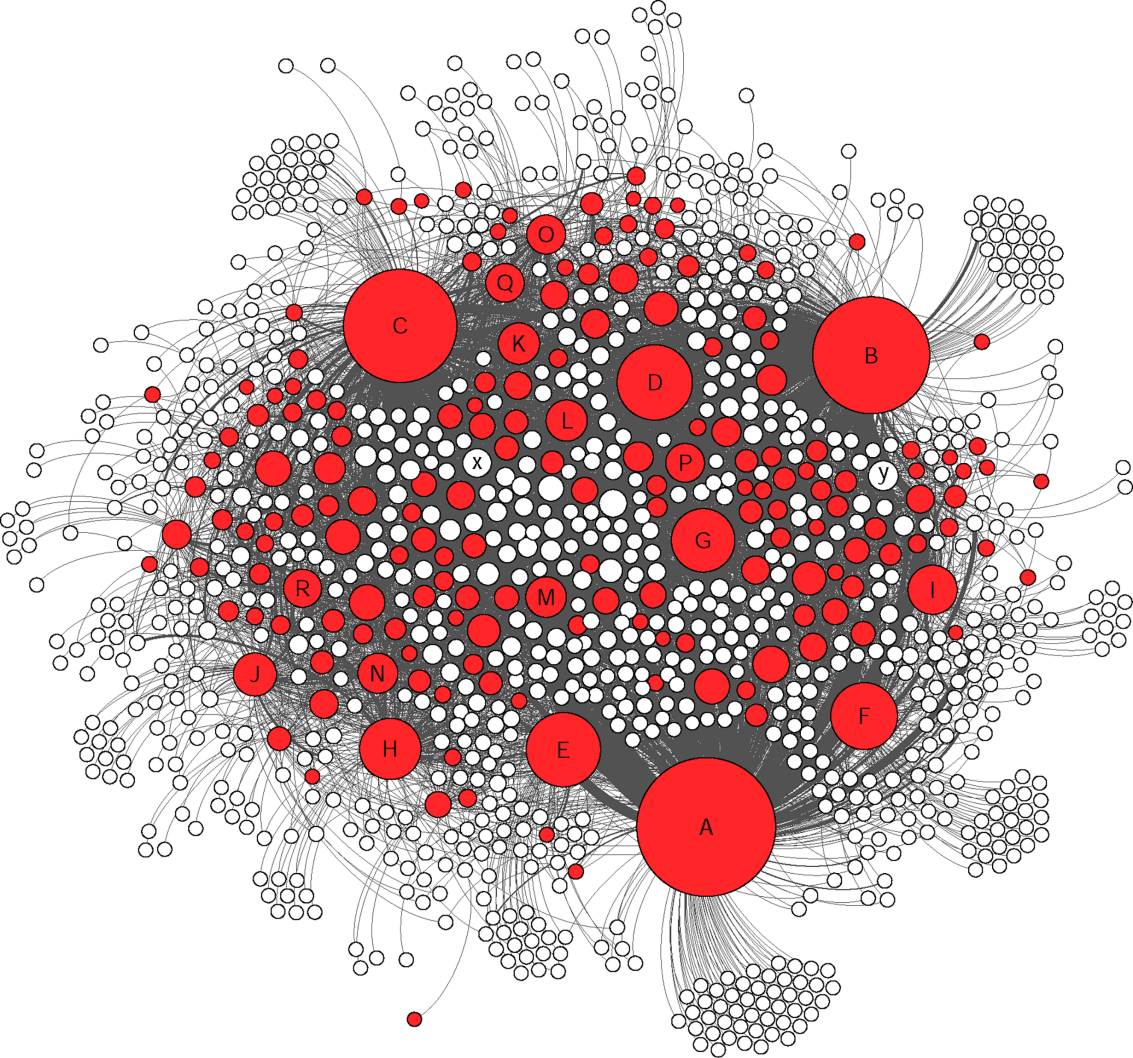
The colored nodes shows the members who were involved in publishing posts (16% of the nodes). The size of each node represents the degree centrality. The marked nodes A-R are well-connected and very-well-connected nodes in the network. The x and y nodes are two other active nodes who just made “comments” and “likes”.

**Fig 4:**
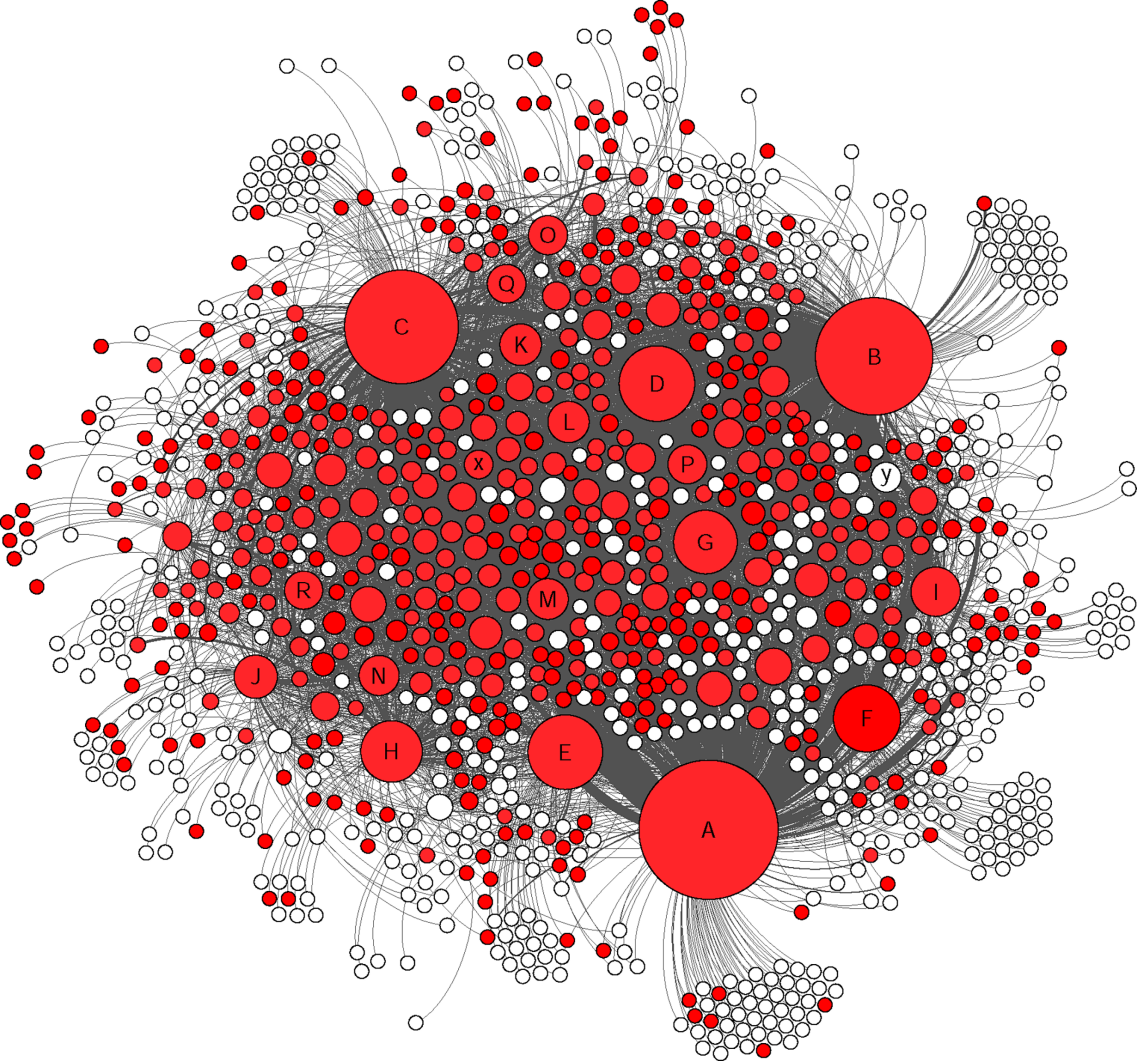
The colored nodes shows the members who were involved in making comments (49% of the nodes). The size of each node represents the degree centrality. The marked nodes A-R are well-connected and very-well-connected nodes in the network. The x and y nodes are two other active nodes who just made “comments” and “likes”.

**Fig 5:**
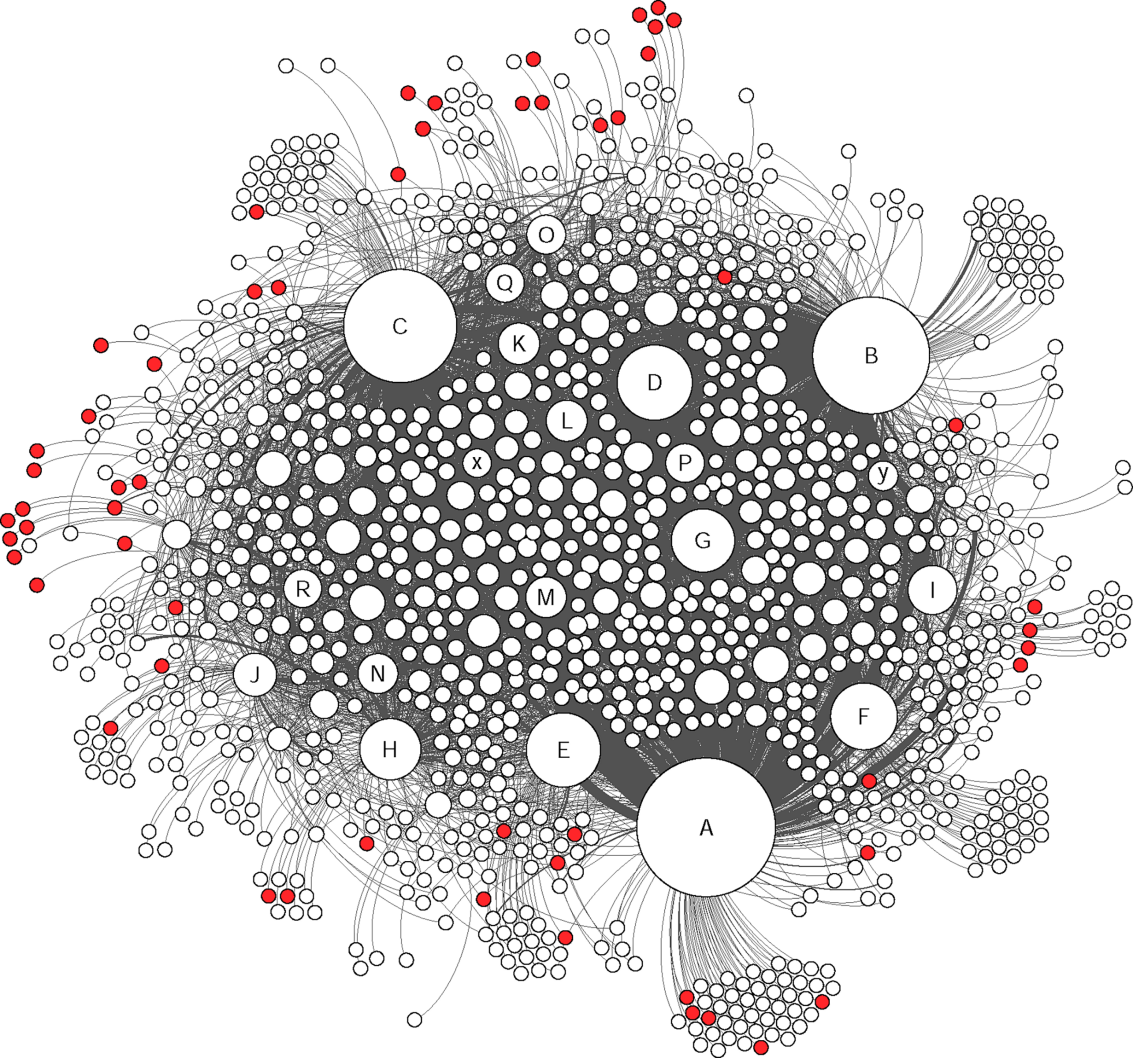
The colored nodes shows the members who were only making comments (5% of the nodes). The size of each node represents the degree centrality. The marked nodes A-R are well-connected and very-well-connected nodes in the network. The x and y nodes are two other active nodes who just made “comments” and “likes”.

**Fig 6:**
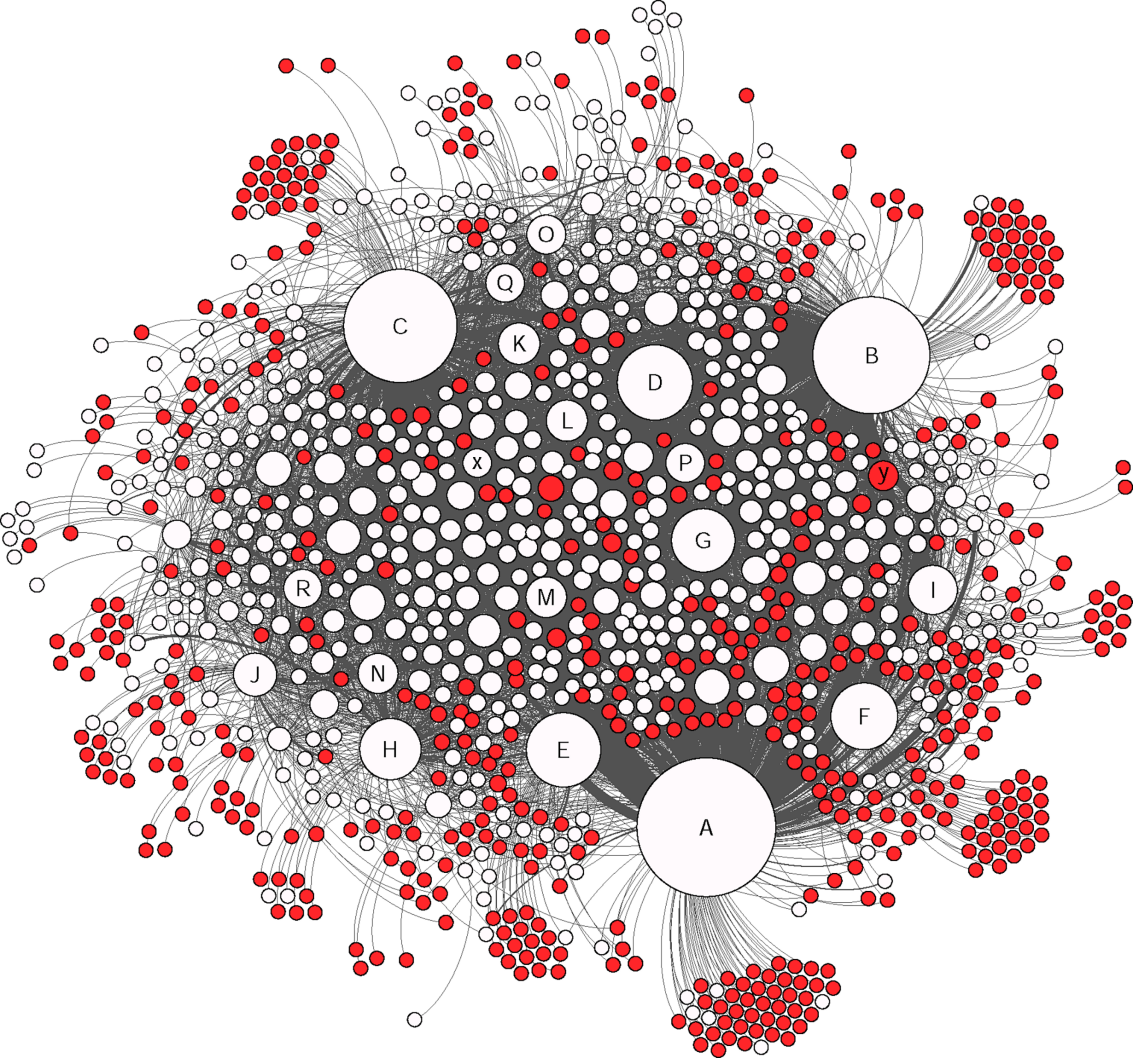
The colored nodes shows the members who were only making likes (48% of the nodes). The size of each node represents the degree centrality. The marked nodes A-R are well-connected and very-well-connected nodes in the network. The x and y nodes are two other active nodes who just made “comments” and “likes”.

The social network analysis provided a clear visualization of how members were connected in this group and the nodes were divided based on their level of connectivity (number of connected nodes) (Fig 2 and Table 6). In this study only direct connections between different nodes were considered and based on their interaction types, different groups of nodes were identified (Figs 3, 4, 5 and 6).

**Table 6:** Nodes division based on connectivity.

Levels of connectivity	Amount of Nodes	Degree centrality	Weighted-centrality	Only made likes	Only made comments	Only made posts
Zero-connected	1 (0%)	0	0	0	0	1 (<1%)
Single-connected	297 (27%)	1	1–10	243 (82%)	41 (2%)	1 (<1%)
Bi-connected	155 (14%)	2	2–7	112 (72%)	12 (1%)	1 (<1%)
Sparsely-connected	381 (35%)	3–10	3–36	85 (22%)	30 (1%)	3 (<1%)
Intermediate-connected	240 (22%)	11–94	11–127	20 (1%)	0 (0%)	3 (<1%)
Well-connected	15 (1%)	100–254	158–534	0 (0%)	0 (0%)	0 (0%)
Very-well-connected	3 (<0%)	411–516	1700–2308	0 (0%)	0 (0%)	0 (0%)

Table 6 shows the division of nodes based on the level of connectivity and different interaction types. In this Facebook group only 18 nodes had more than 100 connections and most nodes were sparsely-connected to 3–10 other nodes followed by single and intermediate-connected nodes (Table 6). Also the nodes with higher degree centrality differed regarding their types of contributions (posts, comments and likes) compared to some of the single, bi and sparsely-connected nodes who had mainly a liker or a commenter personality.

**Fig 7:**
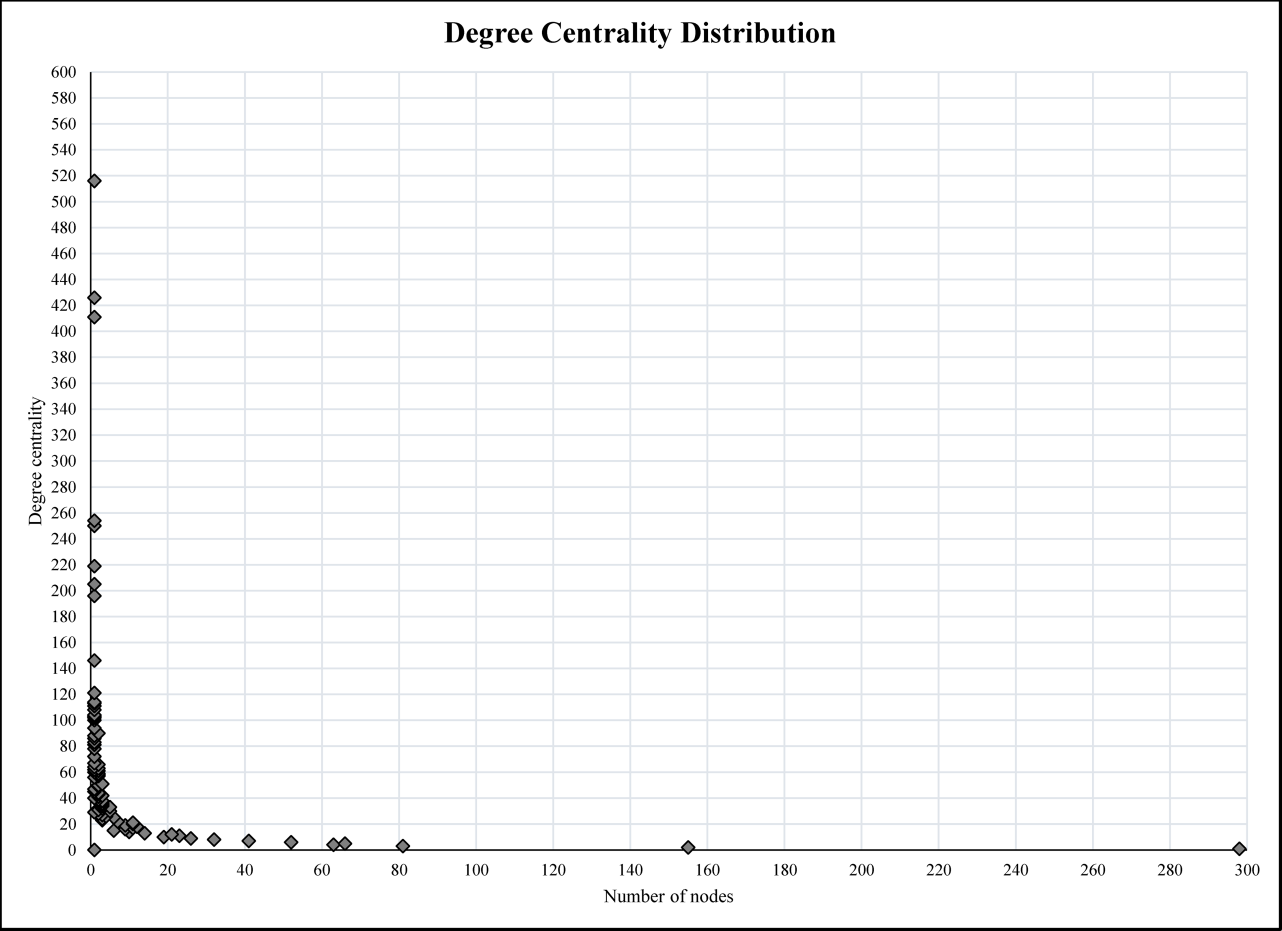
The degree centrality distribution for the whole network.

**Fig 8:**
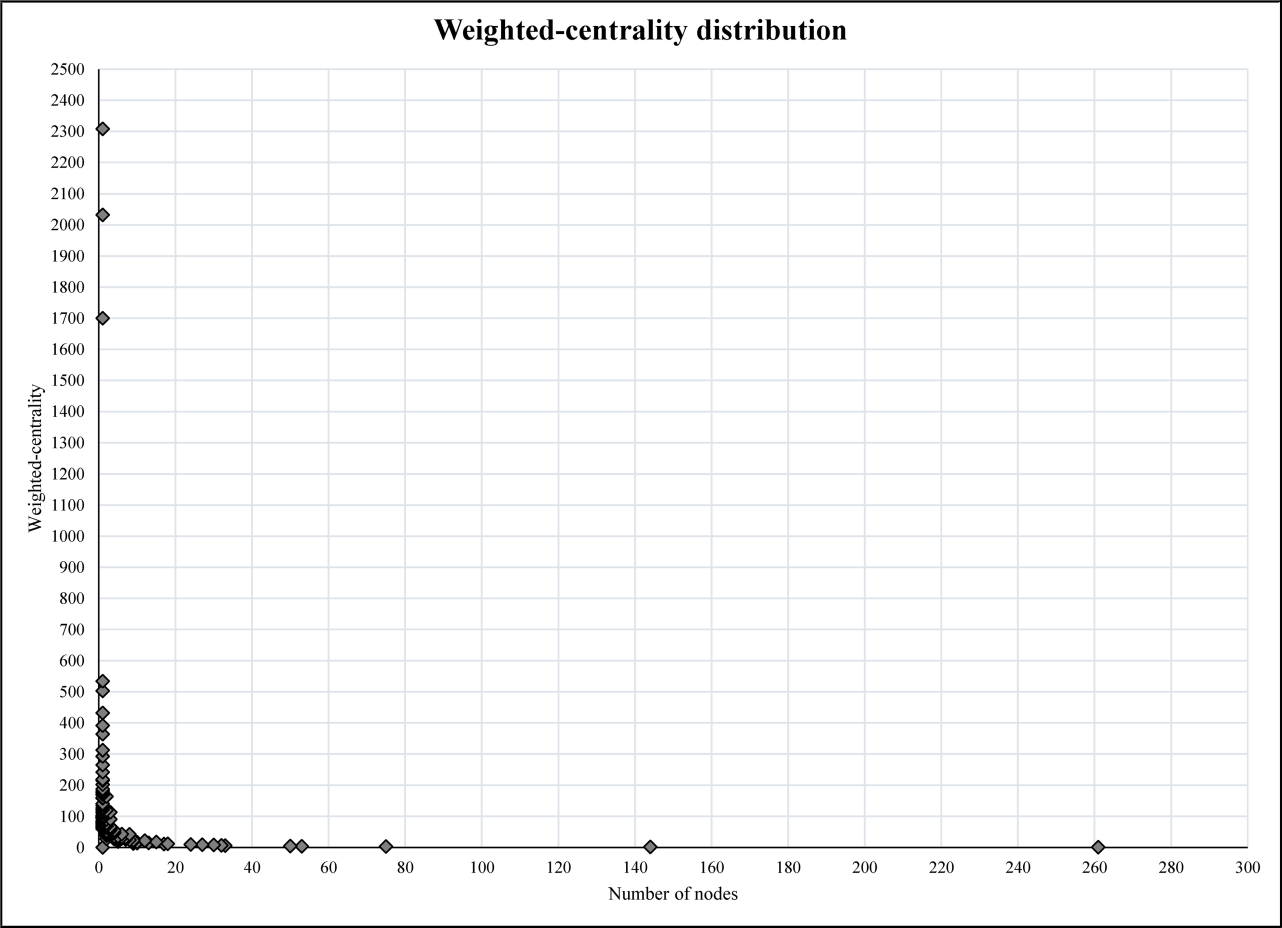
The weighted-centrality distribution for the whole network.

As shown in the Fig 2, the graphs generated from the social network analysis were very nested and the edges between different nodes were difficult to distinguish. However, the density element was not high (0.011 in 0–1 range representing the complete connectivity between the nodes) due to incomplete connectivity within the whole group members. This also showed that only a few nodes were in contact with many others and there were many nodes who were only communicating with one or a few others. Fig 7 shows the degree centrality and Fig 8 weighted-centrality distributions in the group.

To understand the nature of active nodes’ contributions to the group, and different personalities, nodes that had the most contacts in the very well and well-connected categories were marked in the graphs as A-R. Also two other nodes who had an interesting behavior were marked as x and y (Fig 2). The node x was an intermediate-connected node who just made comments and likes towards others without having any posts. The node y’s contribution to the group was only likes. Table 7 shows the details of active nodes’ contribution to the group.

**Table 7:** The numbers of post, comment, likes, connected nodes and directed interactions for selected nodes.

Nodes	Posts	Made comments	Made comments excluding self-comments	Received comments	Made likes	Received likes	Connected nodes (degree centrality)	Directed Interactions (Weighted-centrality)
A	66	332	66	313	110	1970	516	2308
B	82	75	65	114	130	1869	426	2032
C	72	170	50	451	45	1215	411	1700
D	11	88	81	63	75	364	254	534
E	10	59	52	43	96	340	250	503
F	5	1	1	16	7	293	219	312
G	8	35	25	57	36	293	205	391
H	6	46	25	152	24	182	196	364
I	7	15	5	38	4	221	146	265
J	22	22	7	43	7	163	121	215
K	15	11	8	21	4	134	114	160
L	3	88	77	19	156	60	113	293
M	3	140	140	2	249	52	111	432
N	2	9	5	21	49	95	108	164
O	9	34	8	128	2	70	104	202
P	4	15	12	22	25	127	103	170
Q	10	29	24	30	28	101	102	176
R	12	35	17	78	7	67	100	158
x	0	82	82	0	82	0	61	164
y	0	0	0	0	242	0	66	242

**Fig 9:**
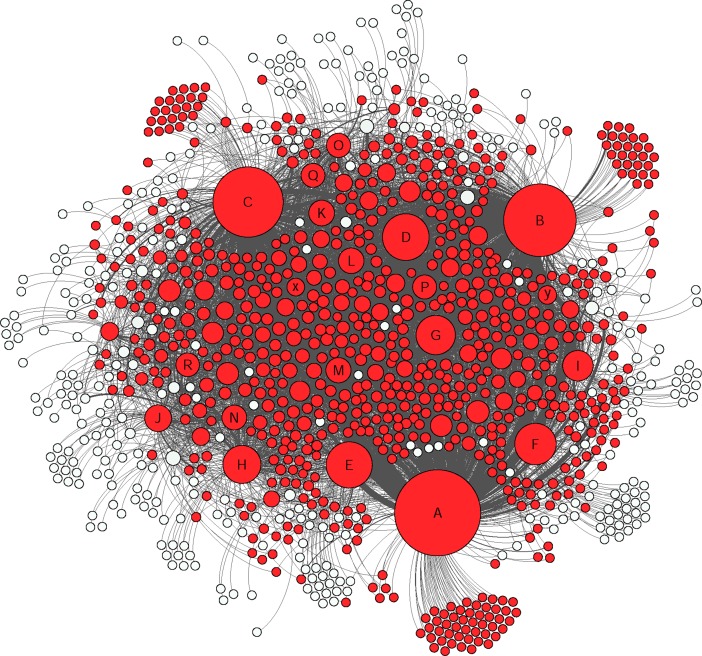
**The proportion of network connected to the nodes A, B and C (colored).** The size of each node represents the degree centrality. The marked nodes A-R are well-connected and very-well-connected nodes in the network. The x and y nodes are two other active nodes who just made “comments” and “likes”.

**Fig 10:**
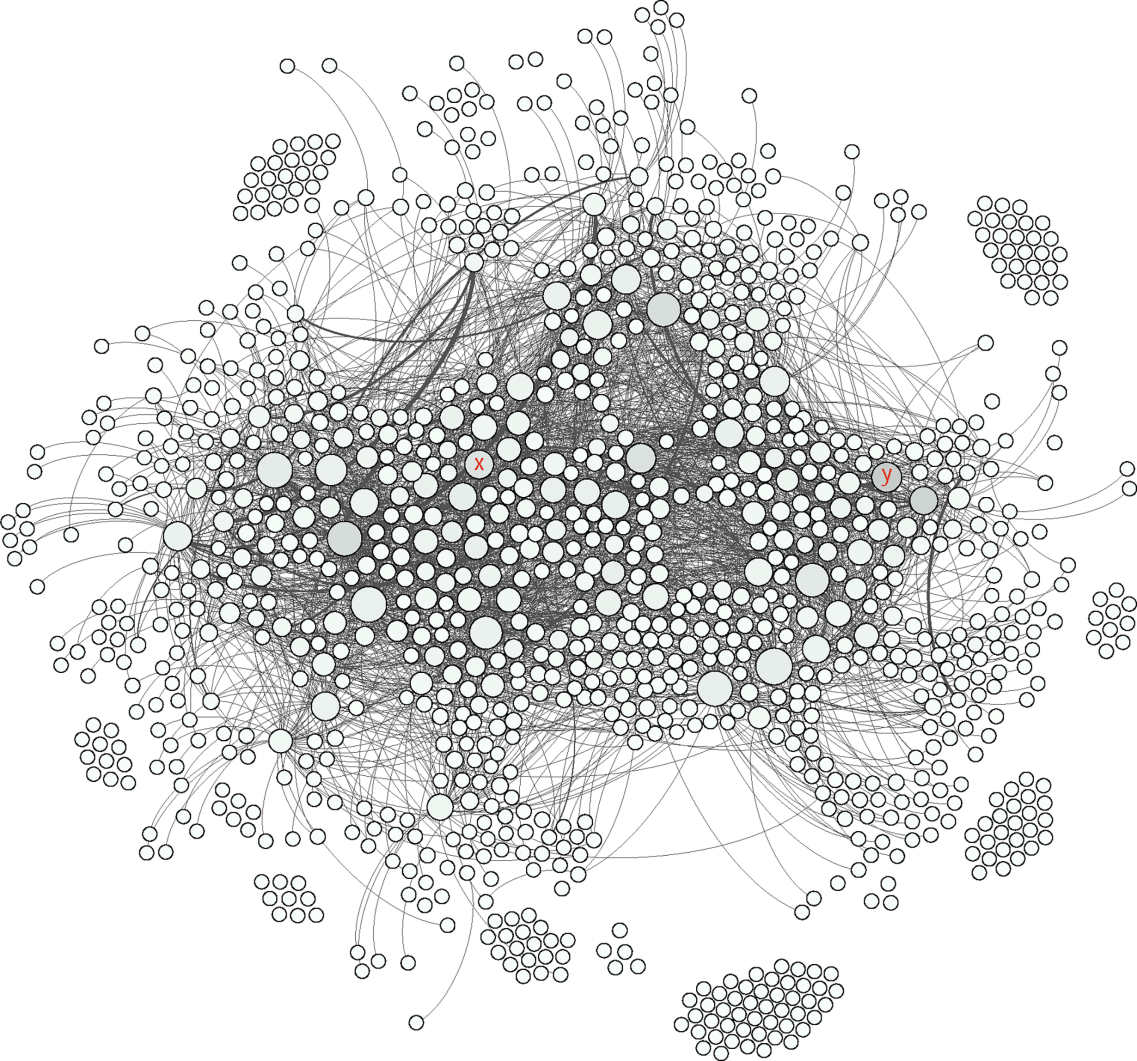
The network nodes and their edges excluding active nodes. The size of each node represents the degree centrality. The x and y nodes are two other active nodes who just made “comments” and “likes”.

The social network analysis showed that the active nodes had their own single-connected followers in addition to other multi-connected nodes (Fig 9). During identification of the most connected nodes, to understand the interaction between other nodes, the very well–and well connected nodes were removed to apprehend the network with less active nodes (Fig 10). As shown in Fig 10, the network between single, bi, sparsely and intermediate nodes is also pretty dense even without active nodes.

In this group, three different types of nodes were identified. The nodes who mostly post in the group, nodes who get into conversation by commenting on the posts and others’ comment, in addition to nodes who just like others’ posts and comments. However, the majority of the nodes (43%) were identified as commenters and likers during the data collection period.

According to Table 7, A, B and C nodes had the highest number of posts in the group in addition to having the highest degree centrality and weighted-centrality in the group (very-well-connected nodes) and as shown in Fig 9, i.e. they have been in contact with a much larger part of the network than the others. Although A, B and C had published the highest amount of posts, they did make highest number of comments and likes per individual. However, they mostly responded to other nodes’ posts than their own published materials. The M, x, D and L nodes made highest amount of comments excluding self-comments. Despite having a small size in the graph, the node M was the most frequent commenter in the group. The x node was chosen to be among this sample due to having the high number of comments but no post or like contribution to the group. This node was only addressing other nodes by responding to already published content instead of posting to the whole group. The most frequent likers in the group were M and y. Regarding receiving comments and likes in posted content, the most frequent receivers of comments were, in order, nodes C, A and H. The node H, was the second most frequent receiver of comments by having very few posts in the group (Table 7).

**Fig 11:**
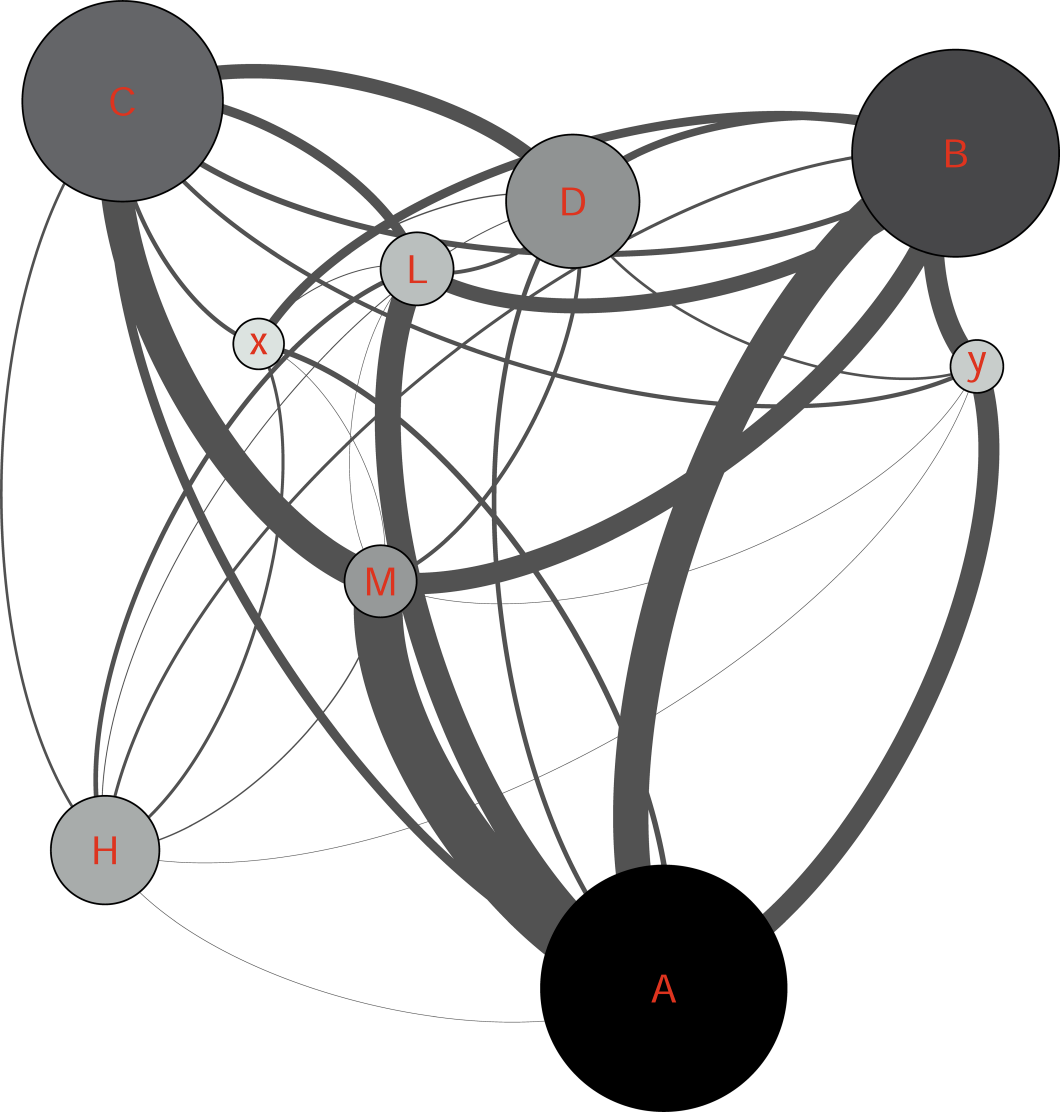
The selected active nodes and their edges. The size of each node represents the degree centrality and the darkness indicates weighted-centrality.

Fig 11 shows that many of the most frequent producers and receivers of comments or “likes” were communicating with each other. The thickness of the lines between different nodes shows the edges between them. This figure shows that the information exchange between M and A was the most frequent in the whole network. Interestingly the node H was communicating with a group of single-connected nodes which were not in contact with other active nodes. Furthermore, the exclusion of the active nodes (A, B, C, D, H, M, L, x and y) also resulted in a highly nested network (density 0.007) demonstrating a good communication within the network.

With the intention of getting deeper into the active nodes’ contributions, the top posters and commenters were selected to analyze the semantics of their posts (Table 8), and made and received comments without considering self-loops (comments on their own posts) (Table 9). Most of the post (except “Emotional support”) were providing support.

**Table 8:** The content analysis of active members’ contributions regarding seeking and providing support categories, identified in the social network analysis.

Members	Seeking Informational	Providing Informational	Seeking Emotional	Providing Emotional	Seeking Esteem	Providing Esteem	Seeking Others	Providing Others	Socializing related
A	0/0	13/346	3/188	2/29	2/131	4/115	0/0	3/43	39/1118
B	0/0	1/30	0/0	5/159	0/0	13/254	0/0	1/20	62/1406
C	2/42	28/314	1/4	7/141	0/0	4/90	0/0	4/79	26/545
D	0/0	4/172	0/0	1/43	0/0	2/33	0/0	1/59	3/57
E	1/4	1/31	0/0	1/30	0/0	0/0	0/0	1/58	6/217
F	0/0	1/23	0/0	0/0	0/0	0/0	0/0	0/0	4/270
G	0/0	6/207	1/63	0/0	0/0	0/0	0/0	1/23	0/0
H	0/0	2/74	3/33	0/0	0/0	0/0	0/0	1/75	0/0
I	0/0	2/37	1/43	0/0	0/0	0/0	0/0	3/86	1/55
J	0/0	2/16	2/15	1/5	0/0	2/32	2/17	9/46	4/32
K	0/0	5/76	0/0	0/0	0/0	0/0	1/6	9/52	0/0
L	0/0	3/60	0/0	0/0	0/0	0/0	0/0	0/0	0/0
M	0/0	3/52	0/0	0/0	0/0	0/0	0/0	0/0	0/0
N	0/0	2/95	0/0	0/0	0/0	0/0	0/0	0/0	0/0
O	2/4	4/42	1/13	0/0	0/0	0/0	0/0	2/11	0/0
P	0/0	3/115	0/0	0/0	0/0	0/0	0/0	1/12	0/0
Q	0/0	9/99	0/0	0/0	0/0	0/0	0/2	1/0	0/0
R	3/6	8/22	1/39	0/0	0/0	0/0	0/0	0/0	0/0
x	-/-	-/-	-/-	-/-	-/-	-/-	-/-	-/-	-/-
All posts	65/270	195/2954	32/577	27/541	5/168	26/529	12/100	93/920	175/4128
All comments	-	-	-	-	-	-	-	-	-

The numbers indicate the proportion of posts/received likes per codes. The “Others” code is a summation of “Other” categories in addition to the “Network support” and “Tangible assistance” categories from SSBC by Cutrona and Suhr [33]. The “Socializing related” code is sum of “Banter”, “Socializing” and “Non-verbal cues” from others categories with no seeking and providing division (Tables 1, 2 and 3).

**Table 9:** The content analysis of active members’ contributions considering different social support categories, identified in the social network analysis.

Members	Informational	Emotional	Esteem	Others	Socializing related
A	3/61	15/54	17/103	7/25	24/70
B	6/8	14/11	18/53	3/9	24/33
C	8/30	12/265	14/49	6/21	10/86
D	24/13	31/7	15/31	5/3	6/9
E	13/4	7/3	5/23	2/2	25/11
F	0/4	0/1	1/8	0/0	0/3
G	4/8	12/22	5/19	3/2	1/6
H	6/58	9/66	3/14	4/8	3/6
I	2/1	0/13	2/21	1/1	0/2
J	6/5	1/16	0/13	0/7	0/2
K	4/4	0/0	1/1	2/10	1/6
L	23/3	13/5	21/5	5/5	15/1
M	41/0	35/0	20/2	15/0	29/0
N	1/2	0/3	1/11	1/3	2/2
O	6/76	1/27	0/14	1/9	0/2
P	8/7	3/4	0/7	0/3	1/1
Q	16/11	3/0	4/5	1/13	0/1
R	6/39	5/27	3/4	2/8	1/0
x	40/-	20/-	14/-	6/0	2/0
All posts	-/-	-/-	-/-	-/-	-/-
All comments	1136/-	883/-	619/-	316/-	392/-

The numbers indicate made/received comments without considering self-loops per codes. The “Others” code is a summation of “Other” categories in addition to the “Network support” and “Tangible assistance” categories from SSBC by Cutrona and Suhr [33]. The “Socializing related” code is sum of “Banter”, “Socializing” and “Non-verbal cues” from others categories with no seeking and providing division (Tables 1, 2 and 3).

Bearing in mind the nodes with the highest number of published posts (A, B and C), their contributions were the most liked in the network and they typically responded to posts from other members. Moreover, in addition to making the highest number of likes, these nodes received the highest number of likes in different support categories. Remarkably, A and B had fewer informational posts than other active nodes in addition to the whole group. Also the C node had a similar amount of posts in different category to the whole group. Regarding influential posts, the H node had a few seeking emotional support posts which received the highest amount of comments per post in the network. None of the active nodes were involved in negative interaction. Also the most frequent commenters’ (M and x) contribution to the group had a similar theme to the whole group overall comments’ categories. Moreover, the C node received the highest number of comments in the “Socializing related” category.

## Discussion

The results of this content and social network analysis concerning persons with brain fatigue after acquired brain injury showed their capacity of using Facebook as a means for communication and support for their condition. However, the lack of data about the participants’ cognitive and general health problems as well as the insecurity about the real identity of persons behind Facebook profiles, needs to be considered.

Indicating the same pattern as other studies for typical Facebook users, the members of this Facebook group acted similar as other Facebook users in general by using features such as likes on posts (as the most common communicative interaction) followed by comments and publishing posts [39,40] (Figs 3, 4, 5 and 6). Group members used posts to address the general public, and comments, to address specific group members. Obviously, the interaction patterns were dependent on the Facebook platform features, thus, the results would not show how this group will use other types of social media such as Twitter, YouTube etcetera [41].

Based on the results (Table 4) and specifically the number of likes per posts, providing any type of support in posts and comments was appreciated by the group members. Similar to other online forums for patients with other health conditions, informational support was a common contribution [33,42]. In addition to providing informational support, banter in posts, and socialization in comments were also quite popular (Table 4). Usually, the informational support would be beneficial for all members and non-members of a group (even the ones who are not a member of the same group) [20] and the provider, will be encouraged and supported by other members’ likes and comments. Regarding the access to information about health, the results replicated the findings by Bunner et al. showing social participation as common reason to use social media for individuals with traumatic brain injury [20]. In this groups, the amount of socializing, non-verbal cues, expression of gratitude and offering congratulation showed that members would appreciate the contact with people in similar situations. Furthermore, the publicness of the group was discussed between group members and some of the members indicated that it should be public to improve the understanding of their condition the among general public. This preference shows social media (in this case Facebook) could be a means to provide a better understanding of persons with specific clinical conditions for the general public in addition to all other potential benefits for its’ own members.

Based on the results (Figs 7 and 8), the degree centrality and weighted-centrality followed a long tail distribution, indicating that the majority of members only communicated with few others and had few direct communications. Bearing in mind the likes and comments per posts, the results also showed that the popularity of active members was dependent on the frequency of communications. Obviously, the members with a higher number of posts had higher degree centrality and weighted-centrality in the network. This finding is in line with other studies on Facebook groups over 1000 members [43]. Unfortunately, the Facebook platform does not show passive members’ activities within the group. In addition, the comment on comment feature on Facebook was not activated during the time of this study.

The results also showed different patterns in content and interaction of a few active individual members of this group. Considering the association of different personalities with self-disclosure and social orientation [44], the members showed different personalities in terms of liking, commenting and publishing posts. However, since we did not have access to their clinical data, no conclusion can be drawn regarding phenomenon being due to the brain injury or a personality trait. Generally, in a real-life support group, these active attention-seeking individuals could take up a lot of space, monopolize other people’s time and use the group for their own purposes [45]. However, in our study removing the active members resulted in a nested network of interactions indicating that if a member did not like one or more active members, they could easily interact with other members and ignore active members and their contribution.

Despite the association of the content analysis results in our study with previous research, none of them focused on different categories in posts and comments nor the number of likes per category. Given the popularity of Facebook for different persons with special health conditions, our methodology could provide a new way to get deeper into unexplored social media features.

Certainly, social media (in this case Facebook) would be a great means of communication and social support for helping persons with MACI. Moreover, most of them with basic knowledge of using ICT tools would be able to use this medium easily on different devices.

### Implication/trustworthiness and patients’ advocacy

Since social contact decreases after cognitive disturbances [21], persons with brain fatigue after brain injury appreciate the information support in communication. This might have an association with the general use of social media which has to be followed in further research. However, it has been known that patients suffering from a mild brain injury show a lower occurrence of mental fatigue symptoms when perceiving social support [46].

In this study, the accuracy and benefits of the exchanged information have not been analyzed. However, the potential risks of inaccurate information and unhealthy behavior are substantial. Noticeably, providing the informational support by most frequent publishers, was highly appreciated and received a high number of likes. Based on previous research, people with cognitive problems may have a tendency to judge information based on being familiar with the source rather than the trustworthiness of it [47]. Therefore, the probability of trusting information provided by active members might be higher than by other information sources. As shown in Fig 9, the A, B and C nodes were in contact with a big portion of the members and nearly covered the whole network. Given the trustworthiness of these members, a suggestion for the healthcare system and patient organizations would be to identify the active users through a social network analysis and voluntarily provide them with basic training to moderate or monitor the group in a way that other members benefit from the knowledge of the effects of brain injury. This idea has to be further explored with clinicians to see the burdens regarding choosing a patient ambassador.

### Limitations

A major concern was to establish the representability of this Facebook group compared to the clinical population. Generally, recognizing mild cognitive impairment is problematic even in elderly populations. Therefore, the manifestation of cognitive impairment is relatively unknown for individuals who are suffering from it and also for healthcare professionals [48,49]. After discussing this issue with MACI rehabilitation professionals’ and medical experts, it seemed that for this type of studies, it is not feasible to achieve a higher level of evidence concerning pathology of the participants in this group. It would be applicable to find the target group in laboratory science but not in the real world. Therefore, this new research area and the methodology should be investigated more. In addition, further studies are needed to replicate this study’s results with persons with similar and different chronic conditions in other Facebook groups to show any disease specific behavior. One of the main limitations of this study was that the data collection platform Facebook does not provide any information about all potential members of the group and their activities (rather than likes, comment and posts), and that the directions of the information (social support) flows were not included. Moreover, the reliability of the results could be biased since this study did not provide any information about the cognitive failure levels of individuals behind the Facebook profiles and their health in general.

There are two possible sources of systematic error in the study: 1) the inclusion of members with cognitive problems due to other than somatic disease and 2) inclusion of non-serious members. However, based on the content analysis, the group administrators excluded supposedly non-serious individuals from the group based on other members questioning their behavior. It must be emphasized that this study was performed on a Facebook group for Swedish speaking persons with brain fatigue and cultural influences might have affected the interaction between the group members. Based on participants’ contribution to the Facebook group and support seeking categories, they had common symptoms with persons with MACI but clinical data is missing [3]. Further studies with more controlled variables would probably provide more reliable results for this specific group.

In this study the intention was to investigate different actors’ behavior in such a community and the majority of the participants had low degree centrality but due to practical and technical reasons, the focus was on participants with the highest degree centrality on individual levels and the behaviors of participants with low degree centrality was not examined. It would be interesting to explore and visualize all members’ activities in terms of themes and content of contributions to the group.

## Conclusion

The aim of this study was to explore the behavior in social media for persons with potential MACI. The results of the content and social network analysis of a public Facebook group addressed the lack of knowledge about the potential use of social media as a means for communication for persons with acquired brain injury and potential MACI.

The results showed different patterns in themes and activities of different individual members compared to the whole group. The most used communication feature of Facebook was likes in form of “thumbs-up” and the most common themes of the members’ contribution in this group were characterized by informational support and socializing. The results of this study showed that the communication behaviors of persons with potential MACI could be similar to the healthy population. However, further studies considering also the cognitive failure levels of individuals would provide more reliable results for this specific group.
